# Calomel as a Panacea

**Published:** 1901-10-19

**Authors:** 


### CALOMEL AS A PANACEA.

All the teachings of the schools go to condemn the hunt for panaceas. On every side we find the leaders in our profession asserting that it is the patient rather than the disease which we ought to consider, and throwing scorn upon those unregenerate outsiders who seek for "cure-alls" and put their faith in methods and so-called "systems" of treatment. Only last week Sir William Church was pouring ridicule upon the head of poor old Bishop Berkeley for having held that tar-water was a cure for all diseases. Sir William very properly added, however, that it was not the laity alone who were thus deluded, and he quoted various ancient instances to prove the truth of his assertion. Perhaps he need not have gone back so far, for the search for "cure-alls" has by no means yet come to an end. Only the other day the members of the British Medical Association gravely met to listen to a paper the author of which strove to prove the virtues of a combination of "calomel, water, heat and quinine" in the treatment of a string of diseases almost as long as that which Bishop Berkeley attached with his tar-water. "Bile," says Dr. Rivière, is the "true pathognomic poison." It is "the product by and through which the liver eliminates noxious materials," and when the hepatic excretion is altered or lessened the intestine becomes infected, and pours into the portal vein bacilli and an over-dose of toxins, which the liver can no longer retain or destroy. . . . Then ensue pernicious infections or typhoid symptoms, according to the degree of liver intoxications. The microbes of acute diseases set to work only after auto-intoxication, which depends, I am convinced, directly on hepatic insufficiency." Here we have a good honest system of pathology, and an equally simple system of therapeutics follows almost as a matter of course. "Purgatives sweep away bile"; and so we find that "enteric fever, dysentery, appendicitis, children's diseases (how sweetly simple is a nosology in which no smaller unit than 'children's diseases' finds a place!) and acute diseases in general are speedily cured by judiciously given calomel, which disintoxicates the whole organism and assures intestinal antisepsis." "Calomel with sodium bicarbonate is always well borne by the most sensitive stomachs. It is never contraindicated. I systematically give to all my fever patients calomel, sodium bicarbonate, āā 0·25 cg., put directly on the tongue at midnight, followed next morning by castor oil beaten up with hot water or 8 grams of magnesia (for an adult). The fever always subsides by the next evening, sometimes on the very morning of the aperient draught." "To prevent the return of fever I always employ quinine, which favours the biliary function, but which requires the action of purgation to eliminate the bile. I cover the patient with warm clothing and make him take hot drinks in abundance, assuring him at the same time, after a slight examination intended only to divert his attention from his symptoms, that his illness is only slight and that his cure will be speedy. I prescribe next morning, after the first stool, warm drinks or weak beef-tea—warm drinks to plethoric, and beef-tea to weak patients."

Among the diseases in which this "calomel, water and heat" treatment is to be used are cholera, infantile cholera, meningitis, post-partum infection, and "all infectious diseases," and it is satisfactory to note that "with this treatment we never see grave symptoms in influenza, eruptive fevers, lung or pleural inflammation, nephritis, and acute diseases." Calomel is never to be prescribed in larger doses than 30 centigrams for an adult and 15 centigrams for a child, and it must always be associated with sodium bicarbonate, and followed by castor oil or other mild aperient five hours later. Next morning the patient must stay in bed, in winter in a warm room, and he must not take ice or cold drinks under any pretence; all of which reminds one greatly of the "pill and possett" treatment of one's early days. After the first stool he must have hot drinks or beef tea for the rest of the day. "It is not true," says Dr. Rivière "that purgatives weaken the patient, as is said so commonly." "All diseases can be referred to one cause—intoxication, and, above all, auto-intoxication, which, in my opinion, depends directly on the disturbance and deficiency of the hepatic function. The human mechanism, like any other machine, will only work when it has been cleaned." Here we have pathology and treatment all in a nutshell, and those who protest against the hunt for panaceas may well raise their voices against such a simplification of the whole duty of the physician. What is the use of medical science if all diseases are to be set right with a pinch of calomel? Nevertheless it is likely enough that in ordinary daily practice the man who uses this all-powerful drug pretty freely, on occasions, will be able to give many points to his competitors who restrict their prescribings to less positive medicaments. The fact is that Dr. Rivière's paper, exaggerated as it no doubt is in certain directions, is a not altogether untimely protest against the modern tendency which seems to drive the physician of to-day to let simple cases drift and to send severe ones to the surgeon.

